# Sensitization Patterns to Aeroallergens and Food Allergens Among Children with Atopic Asthma in Southwestern Saudi Arabia

**DOI:** 10.3390/children12050573

**Published:** 2025-04-29

**Authors:** Ali Alsuheel Asseri, Mashael Abdualslam Abuaqil, Abdulaziz Saud Alotaibi, Wajd Abdualslam Abuaqil, Abdulaziz Saeed Alqahtani, Lama Ali Asiri, Mona Alkhayri, Amal Y. Moshebah, Faten M. ElAbd

**Affiliations:** 1 Department of Child Health, College of Medicine, King Khalid University, Abha 62529, Saudi Arabia; 2 College of Medicine, King Khalid University, Abha 62529, Saudi Arabia; mashaelm391@gmail.com (M.A.A.); 0109az28@gmail.com (A.S.A.); wajd5811@gmail.com (W.A.A.); abdulaziz111sq@gmail.com (A.S.A.); asirilama29@gmail.com (L.A.A.); malkairi@kku.edu.sa (M.A.); 3 Departments of Pediatric, Abha Maternity and Children’s Hospital, Abha 333042, Saudi Arabia; 4 Departments of Serology, Abha Maternity and Children’s Hospital, Abha 333042, Saudi Arabia; elabd3@gmail.com

**Keywords:** bronchial asthma aeroallergens, sensitization, Saudi Arabia, food sensitization

## Abstract

Background and Objectives: Asthma is a prevalent chronic respiratory disease in children, with increasing rates in Saudi Arabia. Allergen sensitization plays a crucial role in asthma development and severity. This study aimed to investigate the prevalence and clinical impact of aeroallergen and food sensitization in children with asthma in Southwestern Saudi Arabia. Materials and Methods: A retrospective chart review was conducted at Abha Maternity and Children’s Hospital, including 194 children aged 3–12 years with atopic asthma. Sensitization to 26 common aeroallergens and food allergens was assessed using the EUROLINE Allergy test. Associations between sensitization patterns, atopic comorbidities (allergic rhinitis and eczema), and asthma-related outcomes (hospitalizations, medication use, and school absenteeism) were analyzed. Results: A high prevalence of sensitization was observed (74.2% for aeroallergens; 56.7% for food allergens). Aeroallergen sensitization was associated with older age (*p* < 0.001), male sex (*p* = 0.026), allergic rhinitis (*p* < 0.001), eczema (*p* = 0.295), and increased asthma morbidity, including hospitalizations (*p* = 0.002) and corticosteroid use (*p* = 0.012). Food sensitization was associated with eczema (*p* < 0.001) but did not significantly impact other asthma outcomes. Poly-sensitization was associated with a higher prevalence of eczema (*p* = 0.003). Dust mite sensitization was a strong independent predictor of severe asthma (adjusted odds ratio = 4.4, 95% CI = 1.7–11.8, *p* = 0.003). Conclusions: This study demonstrates a high prevalence of aeroallergen and food sensitization among children with atopic asthma in Southwestern Saudi Arabia, with distinct sensitization patterns and associated comorbidities. Aeroallergen sensitization, particularly to dust mites, was associated with increased asthma morbidity, highlighting the importance of comprehensive sensitization assessment in this population. While limited by its retrospective design, this study provides valuable insights into the interplay between sensitization and childhood asthma, informing future research and clinical practice.

## 1. Introduction

Asthma is a chronic respiratory disease affecting millions of children worldwide. It is characterized by airway inflammation and hyperresponsiveness, leading to recurrent episodes of wheezing, breathlessness, chest tightness, and cough [1,2,3,4]. The development and severity of asthma are influenced by a complex interplay of genetic and environmental factors, including exposure to allergens [5,6]. Allergen sensitization, mediated by immunoglobulin E (IgE) antibodies, plays a crucial role in triggering and exacerbating asthma symptoms, particularly in children [7,8]. Both aeroallergens (inhalant allergens) and food allergens can contribute to sensitization and asthma morbidity [8].

Understanding the prevalence and clinical impact of sensitization to different allergens is crucial for effective asthma management. Studies have shown that sensitization to specific allergens, such as house dust mites, can be associated with increased asthma severity and healthcare utilization [9,10]. Moreover, sensitization to multiple allergens (poly-sensitization) has been linked to a higher risk of severe asthma and other atopic conditions like eczema [11,12,13,14]. However, there is still a need for more research to fully elucidate the complex relationship between sensitization patterns, atopic comorbidities, and asthma outcomes, particularly in different geographic regions and populations [15].

In Saudi Arabia, the prevalence of asthma and allergic diseases is increasing [16,17]. Several studies have investigated the prevalence of sensitization to specific aeroallergens in the region, identifying house dust mites, animal dander, and pollen as common sensitizers [18]. However, there are limited data on the comprehensive assessment of both aeroallergen and food sensitization and their combined impact on asthma severity and related outcomes in Saudi Arabian children.

Therefore, this study aimed to investigate the prevalence of sensitization to a wide range of aeroallergens and food allergens in a cohort of children with atopic asthma in southwestern Saudi Arabia. We also sought to examine the association between sensitization patterns, atopic comorbidities (allergic rhinitis and eczema), and asthma-related outcomes (hospitalizations, medication use, and school absenteeism). Furthermore, we explored the clinical characteristics and outcomes of children with mono-sensitization versus poly-sensitization. By providing a comprehensive assessment of sensitization profiles and their clinical implications, this study contributes valuable insights into the management of childhood asthma in the region and adds to the growing body of knowledge on the complex interplay between sensitization and asthma.

## 2. Material and Methods

### 2.1. Study Design, Setting, and Population

A retrospective chart review was conducted between October 2024 and January 2025 at the Abha Maternity and Children’s Hospital Asthma Clinic (southwestern Saudi Arabia) to identify children aged 3–12 years with atopic asthma. This hospital is considered a tertiary care and teaching hospital in the southwestern region of the Kingdom of Saudi Arabia. The study included 194 children who met the criteria for a physician diagnosis of asthma [16], clinical confirmation of atopic asthma, and serological evidence of atopy (a high total IgE level (>100 IU/mL) and the presence of serum-specific IgE (sIgE) of 3.5 IU/mL or more to at least one aeroallergen or food allergen).

### 2.2. Asthma Severity Assessment

Asthma severity was classified by the level of medication needed for symptom control, following established guidelines. Mild asthma required low-dose ICS, moderate asthma needed low-dose ICS/LABA or medium-dose ICS, and severe asthma involved moderate- or high-dose ICS/LABA, possibly with montelukast [1]. Additionally, further asthma morbidity was assessed using the following variables: a history of severe exacerbations (defined as prior hospitalization and pediatric intensive care unit (PICU) admission), healthcare utilization (including total hospitalization days and emergency room (ER) visits in the past year), systemic medication use (measured by the number of corticosteroid (CS) courses in the past year), and the impact on daily life (indicated by the median number of school days missed in the past year).

### 2.3. Serological Assessment of Total and Allergen-Specific Serum Immunoglobulin E

An ELISA kit (Human Diagnostics, Germany, catalog number #51015) was used to measure total IgE. Serum-specific IgE antibodies were measured using a semi-quantitative EUROLINE Allergy test (EUROIMMUN, Lübeck, Germany) following the manufacturer’s instructions. Children with confirmed sensitization to at least one aeroallergen or food allergen were included in the study. These participants underwent further testing to determine specific IgE antibody levels against a panel of 26 common inhalant and food allergens. The inhalant allergens tested included indoor allergens (house dust mites [*Dermatophagoides pteronyssinus* and *Dermatophagoides farinae*], animal dander (cat, dog, and horse), and molds (*Cladosporium herbarum*, *Aspergillus fumigatus*, and *Alternaria alternata*) and outdoor allergens (weed pollen [*Artemisia vulgaris*/mugwort and birch] and a grass pollen mix (timothy grass and cultivated rye). The food allergens tested included egg white, egg yolk, cow’s milk, codfish, α-lactalbumin, β-lactoglobulin, casein, bovine serum albumin, wheat, rice, soy, peanut, hazelnut, carrot, potato, and apple. Specific IgE concentrations exceeding 0.35 kUA/L were considered indicative of sensitization.

### 2.4. Statistical Analysis

Data analysis was performed using SPSS version 29 (IBM Corp., Armonk, NY, USA). Descriptive statistics summarize patient characteristics. Continuous variables are presented as mean ± standard deviation or median (interquartile range) based on their distribution. Categorical variables are expressed as frequencies and percentages. Differences between two groups pertaining to continuous variables were assessed using independent samples *t*-tests or Mann–Whitney U tests, as appropriate. Pearson’s chi-square or Fisher’s exact tests were used for categorical variable comparisons. The relationship between sensitization (both inhalant and food) and demographic/clinical characteristics was examined using the aforementioned statistical tests. For the analysis of mono- and poly-sensitization, comparisons between the two groups were performed using similar methods. To identify the independent predictors of severe asthma, univariable and multivariable binary logistic regression models were constructed. The dependent variable was defined as having severe asthma. Independent variables included sensitization status (dust mites, grasses, and others as determined by clinical relevance), gender, age, and food sensitization. Results are presented as odds ratios (ORs) with 95% confidence intervals (CIs). A *p*-value of <0.05 was considered statistically significant.

## 3. Results

### 3.1. Population Characteristics

Table 1 presents the clinical and epidemiological characteristics of the enrolled patients stratified by the sensitization status. A total of 194 patients were enrolled in the study, with 144 (74.2%) exhibiting aeroallergen sensitization and 110 (56.7%) demonstrating food sensitization. The mean age of the participants was 7.5 ± 3.03 years. Children with aeroallergen sensitization were significantly older than those without (8.0 ± 2.7 years vs. 6.2 ± 3.5 years, *p* < 0.001). This age difference was also reflected in the distribution across age categories, with a higher proportion of aeroallergen-sensitized children being 5 years old or older (*p* < 0.001). No significant age difference was observed between children with and without food sensitization (*p* = 0.106). A higher proportion of males were observed in the aeroallergen-sensitized group compared to the non-sensitized group (66.0% vs. 50.0%, *p* = 0.026). No significant sex difference was found between food-sensitized and non-sensitized children (*p* = 0.339). Blood eosinophil counts and total IgE levels were significantly associated with both aeroallergen and food sensitization. Participants sensitized to aeroallergens (*n* = 144, 74.2%) exhibited a significantly higher median (IQR) blood eosinophil count of 400 (260–693) cells/mm^3^ compared to the non-sensitized group’s 254 (130–500) cells/mm^3^ (*p* = 0.009). Similarly, food-sensitized participants (*n* = 110, 56.7%) had a significantly elevated median (IQR) blood eosinophil count of 435 (300–725) cells/mm^3^ compared to the non-food-sensitized group’s 300 (190–500) cells/mm^3^ (*p* < 0.001). Furthermore, median (IQR) of total IgE levels were significantly higher in aeroallergen-sensitized individuals (455 [194–979] IU/mL) compared to their non-sensitized patients (115 [20–505] IU/mL; *p* < 0.001), and also significantly higher in food-sensitized individuals (603 [248–1340] IU/mL) compared to those not sensitized to food (162 [48–432] IU/mL; *p* < 0.001).

### 3.2. Associated Comorbidities and Asthma Severity

The family history of asthma was significantly more common in aeroallergen-sensitized children (75.0% vs. 42.0%, *p* < 0.001), but not in food-sensitized children (*p* = 0.381). Aeroallergen sensitization was significantly associated with a higher prevalence of allergic rhinitis (80.4% vs. 44.0%, *p* < 0.001). Food sensitization was also associated with eczema, with a higher proportion of food-sensitized children exhibiting eczema (92.8% vs. 69.8%, *p* < 0.001). Asthma severity distribution differed significantly based on aeroallergen sensitization. Among aeroallergen-sensitized participants (*n* = 144, 74.2%), a higher proportion had moderate (85.1%, *n* = 86) and severe (87.5%, *n* = 28) asthma compared to non-sensitized individuals (moderate: 14.9%, *n* = 15, *p* = 0.006; severe: 12.5%, *n* = 4, *p* = 0.043). No significant difference in asthma severity distribution was observed between food-sensitized (*n* = 110, 56.7%) and non-food-sensitized participants (*p* > 0.05 for all severity categories). Children with aeroallergen sensitization experienced significantly more hospitalizations for asthma exacerbations compared to those without (38.2% vs. 16.0%, *p* = 0.002). However, the total number of hospitalization days did not differ significantly between the groups (*p* = 0.687). A similar trend was observed for CS use in the past year, with aeroallergen-sensitized children having a higher median number of CS courses (3 vs. 2, *p* = 0.012). Notably, food sensitization was not associated with a significant increase in hospitalizations, hospitalization days, or CS use. While no significant difference was found in the total number of school days missed between aeroallergen-sensitized and non-sensitized children (*p* = 0.467), food-sensitized children missed significantly fewer school days compared to their non-sensitized counterparts (median = 11 days vs. 12.5 days, *p* = 0.045).

### 3.3. Patient Characteristics Stratified by Inhalant Sensitization Status (Mono- and Poly-Sensitized)

Patients were categorized based on inhalant sensitization status: mono-sensitized (*n* = 46, 32%) and poly-sensitized (*n* = 98, 68.1%) (Table 2). While the two groups showed similar distributions in terms of age (mean age = 7.6 vs. 8.2 years, *p* = 0.106), sex (65.2% vs. 66.3% male, *p* = 0.557), and family history of asthma (69.6% vs. 78.6%, *p* = 0.141), a statistically significant difference emerged in the prevalence of eczema (52.2% vs. 72.4%, *p* = 0.003). Although allergic rhinitis was more common in the poly-sensitized group (82.7% vs. 74%, *p*= 0.131), this difference was not statistically significant. A higher proportion of poly-sensitized patients (72.4%) had eczema compared to mono-sensitized patients (52.2%, *p* = 0.003). Asthma severity distribution did not significantly differ between mono-sensitized (*n* = 46, 32.0%) and poly-sensitized (*n* = 98, 68.1%) participants across the mild (45.5% vs. 54.5%, *p* = 0.248), moderate (31.4% vs. 68.6%, *p* = 0.434), and severe (22.4% vs. 78.6%, *p* = 0.134) categories. There were no significant differences between the groups in terms of asthma-related outcomes, including prior hospitalization for exacerbation (39.1% vs. 38.8%, *p* = 0.977), total hospitalization days (median = 13.5 vs. 12 days, *p* = 0.134), ER visits in the past year (median = 3 in both groups, *p* = 0.471), corticosteroid use in the past year (median = 3 in both groups, *p* = 0.522), prior PICU admission (19.6% vs. 17.3%, *p* = 0.764), or school days missed due to asthma (median 13.5 vs. 12 days, *p* = 0.134). Comparing mono-sensitized (*n* = 46, 32.0%) and poly-sensitized (*n* = 98, 68.1%) participants, blood eosinophil counts did not show a statistically significant difference (median [IQR]: 310 [200–722] vs. 434 [300–679] cells/mm^3^, *p* = 0.088). However, total immunoglobulin E levels were significantly higher in the poly-sensitized group (602 [315–1235] IU/mL) compared to the mono-sensitized group (200 [113–390] IU/mL, *p* < 0.001).

### 3.4. Inhalant and Food Allergen Sensitization Profile

**Figure 1:**
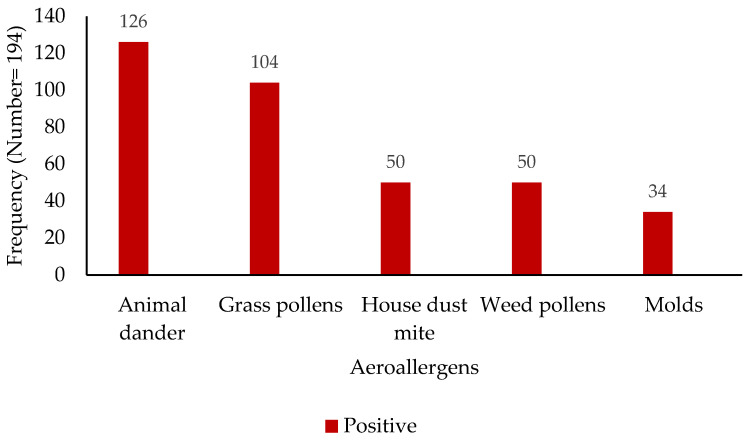
Distribution of aeroallergen sensitization among study participants (*x*-axis—aeroallergen; *y*-axis—patient numbers).

**Figure 2:**
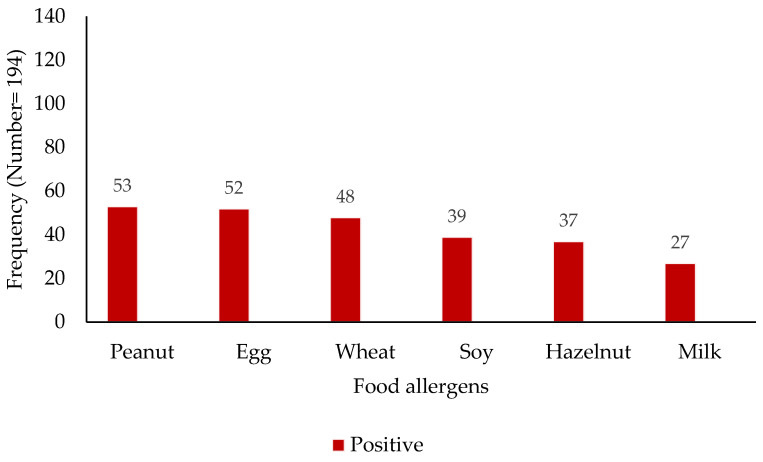
Distribution of food sensitization among study participants (*x*-axis—food allergens; *y*-axis—patient numbers).

The analysis of sensitization profiles revealed distinct patterns across common aeroallergens and food allergens (Figure 1 and Figure 2). Among the assessed aeroallergens, sensitization to animal dander was most prevalent, with 126 subjects (65%) exhibiting a positive reaction, while 68 (35%) tested negative. Grass pollen sensitization was observed in 104 subjects (54%), compared to 90 (46%) who were non-reactive. House dust mite sensitization was identified in 50 individuals (26%), with 144 (74%) showing no sensitization. Similarly, weed pollen sensitization was seen in 50 subjects (26%), with 144 (74%) being non-reactive. Mold sensitization was the least prevalent, with 34 positive reactions (18%) and 160 negative reactions (82%). Regarding food allergens, a spectrum of sensitization was observed. Peanut sensitization was most prevalent (53 positive vs. 142 negative), followed closely by egg (52 positive vs. 142 negative) and wheat (48 positive vs. 146 negative). Soy, hazelnut, and milk sensitization were detected at lower levels: 39 positive vs. 155 negative for soy, 37 positive vs. 157 negative for hazelnut, and 27 positive vs. 167 negative for milk.

### 3.5. Logistic Regression Analysis of Sensitization Predictors of Severe Asthma

Table 3 presents the results of a logistic regression analysis examining the predictors of severe asthma (*n* = 194), and sensitization to dust mites emerged as a significant risk factor. Univariable analysis demonstrated four-fold increased odds of severe asthma in individuals sensitized to dust mites (OR = 4.20, 95% CI = 1.7–10.1, *p* = 0.001). This association remained significant after adjusting gender, age, and food sensitization in a multivariable model (aOR = 4.4, 95% CI = 1.7–11.8, *p* = 0.003). Sensitization to grasses also showed an association with severe asthma in the univariable analysis (OR = 2.60, 95% CI = 1.1–5.9, *p* = 0.027); however, this association was attenuated and was no longer statistically significant after multivariable adjustment (aOR = 2.6, 95% CI = 1.0–6.8, *p* = 0.050). Additionally, sensitization to pollen also showed an association with severe asthma in univariable analysis (OR = 2.40, 95% CI = 1.0–5.4, *p* = 0.041); however, this association was attenuated and was no longer statistically significant after multivariable adjustment (aOR = 2.2, 95% CI = 0.9–5.4, *p* = 0.104).

### 3.6. Logistic Regression Analysis of Sensitization Predictors of ER Visits Last Year

Table 4 presents the results of a logistic regression analysis exploring the predictors of ER visits within the past year (*n* = 194), and sensitization to grasses emerged as a significant risk factor. Univariable analysis revealed a nearly three-fold increase in the odds of an ER visit in individuals sensitized to grasses (OR = 2.90, 95% CI = 1.4–6.0, *p* = 0.004). This association remained statistically significant after adjusting for other potential confounders in a multivariable model (aOR = 2.34, 95% CI = 1.1–5.1, *p* = 0.032).

### 3.7. Logistic Regression Analysis of Sensitization Predictors of Hospitalization

Table 5 presents the results of a logistic regression analysis investigating the predictors of hospitalization (*n* = 194), and sensitization to any aeroallergen was identified as a strong risk factor. Univariable analysis indicated a greater than three-fold increase in the odds of hospitalization among individuals sensitized to any aeroallergen (OR = 3.24, 95% CI = 1.4–7.4, *p* = 0.005). This significant association persisted after adjusting for potential confounders in the multivariable model, demonstrating a nearly four-fold increase in the odds of hospitalization (aOR = 3.70, 95% CI = 1.5–9.0, *p* = 0.004).

Sensitization to dust mites also emerged as a significant predictor of hospitalization. Univariable analysis revealed a more than two-fold increase in the odds of hospitalization in individuals sensitized to dust mites (OR = 2.61, 95% CI = 1.2–5.8, *p* = 0.019). This association remained significant after multivariable adjustment, showing a nearly three-fold increased odds of hospitalization (aOR = 2.80, 95% CI = 1.2–6.5, *p* = 0.018).

Additionally, sensitization to pollen showed a statistically significant association with hospitalization in the univariable analysis (OR = 2.03, 95% CI = 1.1–3.8, *p* = 0.027). However, similar to previous findings, while the trend remained, this association was attenuated in the multivariable model and no longer reached statistical significance (aOR = 2.2, 95% CI = 0.9–4.8, *p* = 0.052).

## 4. Discussion

This study investigated the prevalence and clinical significance of aeroallergen and food sensitization in children with asthma, revealing several key findings. A substantial proportion of the study population exhibited sensitization to both aeroallergens (74.2%) and food allergens (56.7%), highlighting the importance of considering both allergen categories in the management of childhood asthma, particularly the atopic phenotype. Our data demonstrated a significant association between aeroallergen sensitization and age, with aeroallergen-sensitized children being older than non-sensitized children, which aligned with previously published findings [19,20,21]. This finding is consistent with the concept that sensitization may develop over time with increased environmental exposure [22]. The higher prevalence of aeroallergen sensitization in males, observed in our study, warrants further investigation to understand underlying mechanisms. In accordance with the present results, several epidemiologic studies have demonstrated a higher prevalence of atopy among male children compared to female children, as evidenced by positive specific IgE levels or skin prick test (SPT) reactivity to common aeroallergens. A large-scale study conducted in China, investigating the prevalence and distribution of aeroallergen sensitization in children with atopic diseases, reported a significantly higher rate of positive skin SPT results among boys (69.5%, 4218/6071) compared to girls (59.8%, 2067/3456; χ^2^ = 91.7, *p* < 0.01) [23]. This finding suggests a greater propensity for aeroallergen sensitization in male children with atopic conditions. Furthermore, this observation corroborates the findings of AlKhater et al. (2017), who, in a cohort of 100 asthmatic children (86 of whom exhibited confirmed atopy), reported a statistically significant sex difference (*p* = 0.0003), with a predominance of males (*n* = 68) compared to females (*n* = 32) [18]. The higher prevalence of aeroallergen sensitization observed in males within this study may be partially explained by variations in outdoor activity levels. Further research, specifically designed to assess this association, is warranted to more fully explore this hypothesis.

Aeroallergen sensitization was strongly associated with several atopic comorbidities, notably allergic rhinitis and eczema. This is consistent with the established atopic march, where sensitization to environmental allergens can precede or exacerbate these conditions [11,12,14,19,21]. The significant association between food sensitization and eczema, also observed in our study, emphasizes the complex interplay between food and environmental triggers in the development and exacerbation of eczema [24].

Our findings regarding asthma severity are particularly noteworthy. Children with aeroallergen sensitization had moderate/severe asthma and experienced more frequent hospitalizations for asthma exacerbations and a higher median number of CS courses, indicating a greater burden of disease. This underscores the clinical relevance of aeroallergen sensitization in asthma morbidity [25,26]. Two systematic reviews and meta-analyses investigated the association between pollen exposure and childhood asthma exacerbations. The first review, focusing on ER visits, found a statistically significant 1.88% increase in visits per 10 grass pollen grains/m^3^ of exposure, primarily in children aged 5–17. A subsequent review from the same research group, examining hospitalizations, reported a 3% increase in asthma admissions per 10 grass pollen grains/m^3^ of exposure. Collectively, these studies demonstrate a positive association between grass pollen levels and childhood asthma exacerbations requiring both ER visits and hospitalizations [27,28].

Interestingly, while food sensitization was associated with eczema, it did not significantly impact asthma severity, hospitalization rates, or CS use. This suggests that the impact of food sensitization on asthma severity might differ from that of aeroallergen sensitization, possibly through different inflammatory pathways [29,30]. The frequent co-occurrence of asthma and food allergies is a well-documented phenomenon. Epidemiological investigations have established a positive correlation between these conditions [31], suggesting shared pathophysiological mechanisms or common environmental triggers [29,30]. Furthermore, food allergy may either precede the onset of asthma or exacerbate pre-existing asthma symptoms. Schroeder et al. reported a significant association between symptomatic food allergy (FA) and asthma in both older (OR = 4.9, 95% CI = 2.5–9.5) and younger (OR = 5.3, 95% CI = 1.7–16.2) children, with a stronger association in those with multiple/severe FAs, especially in older children [32]. Symptomatic FA was also associated with the earlier onset and greater prevalence of asthma in children with FA compared to those without FA (HR = 3.7, 95% CI = 2.2–6.3 for ≥6 years; HR = 3.3, 95% CI = 1.1–10 for <6 years). Asymptomatic food sensitization showed no association with asthma [32]. However, the precise nature of this association remains a subject of ongoing investigation, with research exploring potential links involving immune dysregulation, genetic predisposition, and environmental exposures. Further research is necessary to fully elucidate the complex interplay between asthma and food allergies and to develop targeted interventions for individuals presenting with both conditions.

When examining inhalant sensitization in more detail, we found a high prevalence of sensitization to animal dander (cat: 47.4%; dog: 9.3%; horse: 8.2%), followed by grass pollen, human dust mites (25.8%) (*Dermatophagoides farina*: 13.9%; *Dermatophagoides pteronyssinus*: 11.9%), and weed pollen. The least prevalent sensitization was to mold. These findings are consistent with other studies conducted in Saudi Arabia and internationally [10,18,33]. Two previous studies in Saudi Arabia offered relevant comparative data for the current findings. AlKhater (2017) [18] found that house dust mites (54%) and cat fur (53%) were the predominant indoor allergens in the eastern region of Saudi Arabia, with Alternaria and Aspergillus (21% each) as the most common mold sensitizers. In contrast, Alqahtani (2016) [34] reported Bermuda grass (43.4%) and cat fur (41.6%) as the most prevalent allergens in the Najran region, with *Dermatophagoides pteronyssinus* (14.8%) and *D. farinae* (10.2%) as the major dust mite allergens. In a study examining the sensitization to common allergens among children with asthma and allergic rhinitis in Qatar, Zahraldin et al. [35] (2021) found that the most common sensitizing allergens were *Dermatophagoides pteronyssinus* (38.1%), *Dermatophagoides farinae* (29.0%), and cat allergen (22.6%). The increase in cat sensitization observed in this study may be attributed to a notable increase in pet ownership, particularly of cats, among the Saudi population [36]. Further investigation is needed to explore the specific factors associated with cat ownership and its impact on sensitization patterns in Saudi Arabia. The significant difference in eczema prevalence between mono- and poly-sensitized individuals, with poly-sensitized children more likely to have eczema, reinforces the concept that multiple sensitizations can contribute to the severity of atopic disease [37].

House dust mite sensitization is a major contributor to respiratory allergies and asthma in children and adults worldwide. High exposure to dust mites can trigger asthma symptoms, while reducing dust mite exposure may improve symptoms. However, studies on dust mite avoidance measures have consistently shown improvements in asthma outcomes, leading to conflicting recommendations in asthma guidelines [38]. A notable finding of this study was the strong association between dust mite sensitization and severe asthma. Even after adjusting for potential confounders, children sensitized to dust mites exhibited over four times the odds of having severe asthma compared to those without sensitization. While univariable analyses suggested a link between grass and pollen sensitization and severe asthma, these associations were attenuated and not statistically significant in the multivariable model. This suggests that the initial association may be partly explained by other factors, such as age, gender, and food sensitization. These findings highlight the importance of dust mite sensitization as a strong, independent predictor of severe asthma.

Our study has some limitations. The retrospective nature of the data collection may have introduced some degree of information bias. The sample size, while adequate for detecting major associations, might have limited our power to detect smaller effects, particularly regarding food sensitization and its impact on asthma severity. Furthermore, the study was conducted at a single center, which may limit the generalizability of our findings to other populations. A notable limitation of this study is the absence of data regarding symptomatic FA, as definitive diagnosis through challenge tests was unavailable. This lack of information restricts our ability to directly correlate observed food sensitization with clinically significant food allergies. Future research should explore these associations in larger, multi-center studies with a prospective design. Longitudinal studies are also needed to understand the temporal relationship between sensitization, atopic comorbidities, and asthma development and progression. Specifically, research should focus on elucidating the mechanisms by which aeroallergen and food sensitization contribute to asthma severity and exploring potential differences in their pathogenic roles. Additionally, investigating the reasons behind the lower school absenteeism in food-sensitized children would be valuable. Finally, understanding the specific regional allergen landscape and its influence on sensitization patterns is crucial for developing targeted prevention and management strategies for childhood asthma. This includes considering both outdoor and indoor allergens and food allergens, as demonstrated by the comprehensive assessment in our study.

## 5. Conclusions

This study demonstrates a high prevalence of aeroallergen and food sensitization among children with asthma in southwestern Saudi Arabia; distinct sensitization patterns and associated comorbidities are also revealed. Aeroallergen sensitization, particularly to dust mites, was associated with increased asthma morbidity, highlighting the importance of comprehensive sensitization assessment in this population. While limited by its retrospective design, this study provides valuable insights into the interplay between sensitization and childhood asthma, informing future research and clinical practice.

## Figures and Tables

**Table 1:** Characteristics of the enrolled patients by sensitization status.

Variable	Total (*n* = 194)	Aeroallergens (*n* = 144, 74.2%)			Food (*n* = 110, 56.7%)		
		Yes	No	*p*-Value	Yes	No	*p*-Value
Age, years, mean ± SD	7.5 ± 3.03	8.0 ± 2.7	6.2 ± 3.5	<0.001	7.3 ± 3.0	7.9 ± 3.0	0.106
Age categories, *n* (%)							
- <5 years	35 (18)	18 (51.4)	17 (48.6)	0.811	25 (71.4)	10 (28.6)	<0.001
- ≥5 years	159 (82)	126 (79.2)	33 (20.8)	<0.001	85 (53.5)	74 (46.5)	0.217
Sex, male, *n* (%)	120 (62.0)	95 (66.0)	25 (50.0)	0.026	70 (63.6)	50 (59.5)	0.339
Family history of asthma, *n* (%)	129 (66.8)	108 (75.0)	21 (42.0)	<0.001	75 (68.2)	54 (65.1)	0.381
Associated atopies for at least one, *n* (%)							
- Allergic rhinitis	137 (70.6)	115 (80.4)	22 (44.0)	<0.001	85 (77.3)	52 (62.7)	0.020
- Eczema	114/136 (83.8)	95/115 (82.6)	19/21 (90.5)	0.295	77/83 (92.8)	37/53 (69.8)	<0.001
Asthma severity, *n* (%)							
- Mild	14/147 (9.5)	11 (78.6)	3 (21.4)	0.491	10 (71.4)	4 (28.6)	0.192
- Moderate	101/147 (68.7)	86 (85.1)	15 (14.9)	0.006	60 (59.4)	41 (40.6)	0.383
- Severe	32/147 (21.8)	28 (87.5)	4 (12.5)	0.043	19 (59.4)	13 (40.6)	0.447
≥1 hospitalization for asthma exacerbation (ever), *n* (%)	63 (32.5)	55 (38.2)	8 (16.0)	0.002	40 (36.4)	23 (27.4)	0.121
Number of total hospitalization days, median (IQR)	12 (9–21)	12 (9–21)	13 (5–16)	0.687	12 (8–21)	13 (9–21)	0.450
ER visits last year, median (IQR)	3 (2–4)	3 (2–4)	2 (1.5–3)	0.069	3 (2–4)	3 (2–4)	0.222
CSs taken last year, median (IQR)	3 (2–4)	3 (2–4)	2 (1.25–3)	0.012	3 (2–4)	3 (2–4)	0.387
Any prior PICU admission, *n* (%)	31/135 (23.0)	25/115 (21.7)	6/20 (30.0)	0.292	19/82 (23.2)	12/53 (22.6)	0.558
School days missed last year, days, (total = 633 days) median (IQR)	12 (8–14.5)	12 (8–15)	10 (6.5–14.25)	0.467	11 (7–13.5)	12.5 (11–16.75)	0.045
Blood eosinophil count, cells/mm^3^, median (IQR)	370 (200–660)	400 (260–693)	254 (130–500)	0.009	435 (300–725)	300 (190–500)	<0.001
Total IgE IU/mL, median (IQR)	379 (139–863)	455 (194–979)	115 (20–505)	<0.001	603 (248–1340)	162 (48–432)	<0.001

SD: standard deviation; IQR: interquartile range; ER: emergency room; CSs: corticosteroids; PICU: pediatric intensive care unit. *p*-value < 0.05 is statistically significant.

**Table 2:** Characteristics of the enrolled patients by inhalant sensitization status, i.e., mono-sensitized and poly-sensitized.

Variable	Mono-Sensitized (*n* = 46, 32.0%)	Poly-Sensitized (*n* = 98, 68.1%)	*p*-Value
Age, years, mean ± SD	7.6 ± 2.9	8.2 ± 2.7	0.106
Age categories, *n* (%)			
- <5 years	7 (15.2)	12 (12.2)	0.104
- ≥5 years	39 (84.7)	86 (87.7)	0.110
Sex, male, *n* (%)	30 (65.2)	65 (66.3)	0.557
Family history of asthma, *n* (%)	32 (69.6)	77 (78.6)	0.141
Associated atopies for at least one, *n* (%)			
- Allergic rhinitis	34 (74.0)	81 (82.7)	0.131
- Eczema	24 (52.2)	71 (72.4)	0.003
Asthma severity, *n* (%)			
- Mild	5 (45.5)	6 (54.5)	0.248
- Moderate	27 (31.4)	59 (68.6)	0.434
- Severe	6 (22.4)	22 (78.6)	0.134
≥1 hospitalization for asthma exacerbation (ever), *n* (%)	18 (39.1)	38 (38.8)	0.555
Number of total hospitalization days, median (IQR)	13.5 (10.25–17.75)	12 (7–13)	0.134
ER visits last year, median (IQR)	3 (2–4)	3 (2–4)	0.471
CSs taken last year, median (IQR)	3 (2–4)	3 (2–4)	0.522
Any prior PICU admission, *n* (%)	9 (19.6)	17 (17.3)	0.497
School days missed last year, days, (total = 633 days) median (IQR)	13.5 (10.25–17.75)	12 (7–13)	0.134
Blood eosinophil count, cells/mm^3^, median (IQR)	310 (200–722)	434 (300–679)	0.088
Total IgE IU/mL, median (IQR)	200 (113–390)	602 (315–1235)	<0.001

SD: standard deviation; IQR: interquartile range; ER: emergency room; CSs: corticosteroids; PICU: pediatric intensive care unit; IgE: immunoglobulin E. *p*-value < 0.05 is statistically significant.

**Table 3:** Logistic regression of unadjusted and adjusted odds ratios for sensitization predictors of patients with severe asthma (N = 194).

Variables	Comparison	Univariable			Multivariable		
		OR	95% CI	*p*-Value	aOR	95% CI	*p*-Value
Sensitization to dust mites	Yes vs. no	4.20	1.7–10.1	0.001	4.4	1.7–11.8	0.003
Sensitization to grasses	Yes vs. no	2.60	1.1–5.9	0.027	2.6	1.0–6.8	0.050
Sensitization to pollen	Yes vs. no	2.40	1.0–5.4	0.041	2.2	0.9–5.4	0.104

The multivariable logistic regression model was adjusted for gender, age, and food sensitization. OR: odds ratio; aOR: adjusted odd ratio; CI: confidence interval. Values of *p* < 0.05 are statistically significant.

**Table 4:** Logistic regression of unadjusted and adjusted odds ratios for sensitization predictors of patients with emergency room visits last year (N = 194).

Variables	Comparison	Univariable			Multivariable		
		OR	95% CI	*p*-Value	aOR	95% CI	*p*-Value
Sensitization to grasses	Yes vs. no	2.90	1.4–6.0	0.004	2.34	1.1–5.1	0.032

The multivariable logistic regression model was adjusted for gender, age, and food sensitization. OR: odds ratio; aOR: adjusted odd ratio; CI: confidence interval. Values of *p* < 0.05 are statistically significant.

**Table 5:** Logistic regression of unadjusted and adjusted odds ratios for sensitization predictors of hospitalization of patients (N = 194).

Variables	Comparison	Univariable			Multivariable		
		OR	95% CI	*p*-Value	aOR	95% CI	*p*-Value
Sensitization to any aeroallergen	Yes vs. no	3.24	1.4–7.4	0.005	3.70	1.5–9.0	0.004
Sensitization to dust mites	Yes vs. no	2.61	1.2–5.8	0.019	2.80	1.2–6.5	0.018
Sensitization to pollen	Yes vs. no	2.03	1.1–3.8	0.027	2.2	0.9–4.8	0.052

The multivariable logistic regression model was adjusted for gender, age, and food sensitization. OR: odds ratio; aOR: adjusted odd ratio; CI: confidence interval. Values of *p* < 0.05 are statistically significant.

## Data Availability

The corresponding author will provide the datasets used and/or analyzed during this study upon reasonable request.
